# Comparison of antifungal and cytotoxicity activities of titanium dioxide and zinc oxide nanoparticles with amphotericin B against different *Candida* species: In vitro evaluation

**DOI:** 10.1002/jcla.23577

**Published:** 2020-09-12

**Authors:** Shima Ahmadpour Kermani, Samira Salari, Pooya Ghasemi Nejad Almani

**Affiliations:** ^1^ Clinic laboratory Social Security Organization Baft Kerman Iran; ^2^ Medical Mycology and Bacteriology Research Center Kerman University of Medical Sciences Kerman Iran; ^3^ Leishmaniasis Research Center Kerman University of Medical Sciences Kerman Iran

**Keywords:** amphotericin B, *Candida* spp, MTT, titanium dioxide nanoparticles, zinc oxide nanoparticles

## Abstract

**Background:**

*Candida* species are known to cause serious fungal infections that produce cutaneous, mucosal, and systemic infections. Nowadays, mortality and morbidity candidiasis in immunocompromised patients have increased. Nanotechnology is a new world‐known technology and includes particles ranging from about 1 to 100 nanometers. The purpose of this study was to evaluate the antifungal and cytotoxicity activities of titanium dioxide nanoparticles (TiO2‐NPs) and zinc oxide nanoparticles (ZnO‐NPs) compared to amphotericin B (AmB) on different *Candida* spp in in vitro conditions.

**Methods:**

In the present study, susceptibility of different *Candida* species to TiO2‐NPs and ZnO‐NPs compared to AmB was determined by broth microdilution (BMD) and agar well diffusion methods. Cytotoxicity of TiO2‐NPs and ZnO‐NPs and amphotericin B was measured by MTT (3‐(4, 5‐Dimethylthiazol‐2‐yl)‐2, 5‐Diphenyltetrazolium Bromide) assay.

**Results:**

The results indicated that the TiO2‐NPs and ZnO‐NPs showed antifungal activities against pathogenic *Candida* spp. The minimum inhibitory concentration (MIC) and minimum fungicidal concentration (MFC) of TiO2‐NP ranges against *Candida* spp. were 128‐256 µg/mL and 256‐512 µg/mL, respectively. The MIC and MFC values of ZnO‐NPs were 64‐128 µg/mL and 256‐512 µg/mL, respectively. However, MICs and MFCs of AmB were 8‐16 µg/mL and 16‐32 µg/mL, respectively. The MTT assay results showed that the CC50% belonged to ZnO‐NPs 706.2 μg/mL, for TiO2‐NPs 862.1 μg/mL, and for AmB 70.19 μg/mL, respectively.

**Conclusion:**

Our findings showed that TiO2‐NPs and ZnO‐NPs had antifungal effects against all *Candida* species, yet the antifungal properties of TiO2‐NPs and ZnO‐NPs were significantly less than those of AmB. The CC50% of AmB was significantly lower than ZnO‐NPs and TiO2‐NPs.

## Graphical abstract

In the present study, susceptibility of different *Candida* species to TiO2‐NPs and ZnO‐NPs compared to AmB was determined by broth microdilution (BMD) and agar well diffusion methods. Cytotoxicity of TiO2‐NPs and ZnO‐NPs and amphotericin B was measured by *MTT* (3‐(4, 5‐Dimethylthiazol‐2‐yl)‐2, 5‐Diphenyltetrazolium Bromide) assay. The results indicated that the TiO2‐NPs and ZnO‐NPs showed antifungal activities against pathogenic *Candida* spp. The minimum inhibitory concentration (MIC) and minimum fungicidal concentration (MFC) of TiO2‐NPs ranges against *Candida* spp. were 128‐256 µg/mL and 256‐512 µg/mL, respectively. The MIC and MFC values of ZnO‐NPs were 64‐128 µg/mL and 256‐512 µg/mL, respectively. MICs and MFCs of AmB were 8‐16 µg/mL and 16‐32 µg/mL, respectively. The MTT assay results showed that the CC50% belonged to ZnO‐NPs 706.2 μg/mL, for TiO2‐NPs 862.1 μg/mL, and for AmB 70.19 μg/mL, respectively.

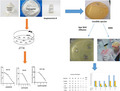

**Abbreviations**
AmB: amphotericin B
BMD: broth microdilution
CC50%: 50% cytotoxic concentration
CLSI: Clinical and Laboratory Standards Institute
DMSO: dimethyl sulfoxide
FLC: fluconazole
MFC: minimum fungicidal concentration
MIC: minimum inhibitory concentration
MTT: 3‐ (4, 5‐Dimethylthiazol‐2‐yl)‐2, 5‐Diphenyltetrazolium Bromide
SDA: Sabouraud dextrose agar
TiO2‐NPs: titanium dioxide nanoparticles
ZnO‐NPs: zinc oxide nanoparticles

## 1 INTRODUCTION

Candidiasis is an opportunistic fungal disease with a great variety of clinical symptoms that can affect various body parts including the skin, nails, oral mucosa, vagina, and internal organs.^1^ These infections can be fatal depending on the host's immune system. This infection is known as one of the major important causes of death, particularly among immunocompromised hosts.^2^ Predisposing factors of *Candida* species include avitaminosis, obesity, pregnancy, alcohol consumption, broad‐spectrum antibiotics and corticosteroids, physiological changes, and age.^3^ Several species of *Candida* such as *C albicans* and non‐*albicans Candida* (NAC) species including *C tropicalis*, *C kefyr, C krusei*, *C glabrata,* and *C parapsilosis* are known as the common cause of candidiasis.^4^ Different species of *Candida* are able to convert to invasive pathogens when the immune system is weakened or the microbial balance of the normal flora of the body is disturbed.^5^, ^6^ Topical antifungal drugs such as nystatin, clotrimazole, and miconazole are used to treat superficial candidiasis. For systemic or disseminated Candidiasis, oral ketoconazole, oral and intravenous fluconazole (FLC), and intravenous amphotericin B (AmB) are recommended.^7^, ^8^

A major concern of the increasing consumption of chemical drugs like antifungal agents is their side effects, which might in some cases be even more dangerous than the disease itself. Headache, nausea, vomiting, hepatotoxicity, and anaphylaxis are some side effects of these drugs.^9^ Other side effects of antifungal drugs include the development of resistant fungal species and therapeutic failures.^1^ For these reasons, many studies have been conducted to find new compounds with antifungal effects.^10^ They are one of the compounds of nanoparticles. The nanoparticles have been studied both individually and in combination with antifungal drugs to achieve better methods for treating various diseases. Furthermore, the nanoparticles are easily available, more affordable, and more effective with similar effects, which makes them a good alternative to chemical drugs.^11^

Titanium dioxide (TiO2) is a nanoparticle, which has been investigated more than any other substance due to its very high photocatalytic activity.^11^ It exists in three crystalline phases including anatase, rutile, and brookite the most stable of which, at normal pressure and temperature, is the anatase structure and the other two phases are semi‐stable.^12^ Some properties of this material that make it preferable to other particles include its high chemical resistance, non‐toxicity, endurance, availability, and low production cost.^13^ In recent decades, the using of zinc oxide (ZnO‐NPs) has increased due to large energy band gaps, chemical‐thermal stability, high oxidation dependence (60 mV), and non‐toxicity.^14^

MTT assay is used to determine the rate of cell proliferation and cell viability, and its mechanism depends on the decrease of insoluble crystals of MTT formazan by the enzyme succinate dehydrogenase in the mitochondria of the cell. Dimethyl sulfoxide (DMSO) solution was used to dissolve these crystals. Then, the amount of light absorption was measured in terms of the intensity of the blue color of formazan at a wavelength of 540 nm. If the cell is alive and reproducing, the rate of dye production and the amount of absorption read are higher, while if more cells are dead and inactive, the rate of light absorption is lower.^15^ The aim of this study was to evaluate the antifungal and cytotoxicity activities of titanium dioxide nanoparticles and zinc oxide nanoparticles compared to amphotericin B.

## 2 MATERIALS AND METHODS

### 2.1 Preparation and determination of Characterization of TiO2‐NPs and ZnO‐NPs

TiO2‐NP and ZnO‐NP powders were purchased from Iranian Nanomaterial Pioneer Company (Mashhad, Iran). Characterization of TiO2‐NPs and ZnO‐NPs was determined by scanning electron microscope (SEM), UV spectroscopy, and X‐ray diffraction (XRD). The SEM micrographs demonstrated the shapes and sizes of the TiO2‐NPs and ZnO‐NPs. The crystalline structure of these nanoparticles was measured using X‐ray diffraction (XRD) (Panalytical, Almelo, Netherlands).

### 2.2 *Candida* spp. and growth condition

This study was carried out on five different *Candida* species isolated from patients with different types of candidiasis. These species were previously identified by real‐time PCR High Resolution Melting Analysis and sequencing methods.^6^ The species included *C tropicalis*, *C parapsilosis*, *C krusei, C albicans*, and *C lusitaniae*. First, the *Candida* spp was subcultured onto Sabouraud dextrose agar (SDA) (Liofilchem Company, Italy).

### 2.3 In vitro antifungal susceptibility testing

#### 2.3.1 Broth microdilution (BMD) method

To determine MIC and MFC values of TiO2‐NPs and ZnO‐NPs compared to AmB on five different *Candida* species, the guideline of Clinical and Laboratory Standard Institute (CLSI, M27‐ED4 document) was followed.^16^ MIC was determined by broth microdilution and agar well diffusion methods.^10^, ^17^ For broth microdilution method, serial dilutions of 4096‐8 μg/mL for TiO2‐NPs and ZnO‐NPs and 128‐0.125 μg/mL for AmB were prepared in a 96‐well microplate containing RPMI‐1640 medium (Sigma‐Aldrich, USA). Then, a suspension containing 1.5 × 10^3^ cells/mL of each *Candida* species was added to all wells. The microplates were incubated in shaker incubator at 35°C for 24 hours. After incubation, each well was compared with the controls and the results as MIC were recorded. MIC values were the lowest concentrations of the TiO2‐NPs, ZnO‐NPs, and AmB, which inhibited the growth of *Candida* by 90% compared to the growth of control. Here, AmB was used as positive control. To measure MFC, 10 µL from wells with no turbidity were cultured on SDA. The plates were incubated at 35°C for 24 hours. After incubation, the lowest concentrations of TiO2‐NPs, ZnO‐NPs, and AmB, with three or less *Candida* colonies, were reported as MFC values.^17^ All experiments were carried out in triplicate.

#### 2.3.2 Agar well diffusion method

The inhibitory effects of various concentrations of TiO2‐NPs, ZnO‐NPs (8192, 4096, 2048, 1024, 512, 256, 128, 64, 32, 16, and 8 μg/mL), and AmB (128, 64, 32, 16, 8, 4, 2, 1, 0.5, 0.25, and 0.125 μg/mL) were evaluated on *Candida* species. A suspension containing 1.5 × 10^3^ cells/mL of each *Candida* species was cultured on Sabouraud dextrose agar medium. Then, 6‐mm wells were created inside the Sabouraud dextrose agar medium by a special punch. Various concentrations of TiO2‐NPs, ZnO‐NPs, and AmB were added to the wells. A well containing distilled water (DD) was considered as a negative control. The plates were placed in an incubator at 35°C for 24 hours. After incubation, the diameters of zones of inhibition of TiO2‐NPs, ZnO‐NPs, and AmB for each *Candida* spp were measured. The tests were performed three times, and the average of the diameters of zones of inhibition of TiO2‐NPs, ZnO‐NPs, and AmB was recorded.

### 2.4 Measuring cytotoxicity of TiO2‐NPs and ZnO‐NPs compared to AmB by MTT assay

MTT assay was used to determine cell proliferation and viability of macrophage J/774.^15^ 1 × 10^4^ mouse macrophages J/774 were cultured in a 96‐well plate. After 24 hours, the culture medium was altered. Then, 10 µL of TiO2‐NPs and ZnO‐NPs at concentrations of 81920‐320 µg/mL and AmB at concentrations of 1280‐2.5 µg/mL was added to mouse macrophages J/774. Then, mouse macrophages J/774 were incubated at 37°C, 5% CO2 and 95% air. After 72 hours of incubation, the conditioned medium was discarded, and 25 μL of MTT solution (Sigma Chemical Co., Germany) was added to each well and incubated in the dark for 3 hours. After this time, the medium containing MTT was carefully removed and 100 µL of DMSO (Merck, Germany) was added to each well of the plate. Finally, the optical absorption was calculated based on the intensity of blue‐colored formazan using a 96‐well microplate reader (BioTek, USA) at 540 nm. If the mouse macrophages J/774 are viable and proliferating, the color is consequently more intense and higher absorbance readings are obtained. The experiment was repeated three times. The concentration of TiO2‐NPs, ZnO‐NPs, and AmB that inhibits mouse macrophages J/774 growth by up to 50% is considered as CC50%. The survival rate of mouse macrophages was calculated using the following formula:

$$\text{Cell}\;\text{survival}\;\text{rate}=\text{Adsorption}\left(\text{OD}\right)\text{of}\;\text{treated}\;\text{sample}/\text{Adsorption}\left(\text{OD}\right)\text{control}*100$$

Adsorption (OD) in treated samples including mouse macrophages J/774 treated with ZnO‐NPs, TiO2‐NPs and AmB was compared with OD levels in control group containing the mouse macrophages J/774 grown in medium without ZnO‐NPs, TiO2‐NPs, and AmB.

## 3 RESULTS

### 3.1 Characterizations of TiO2‐NPs and ZnO‐NPs

**Figure 1:**
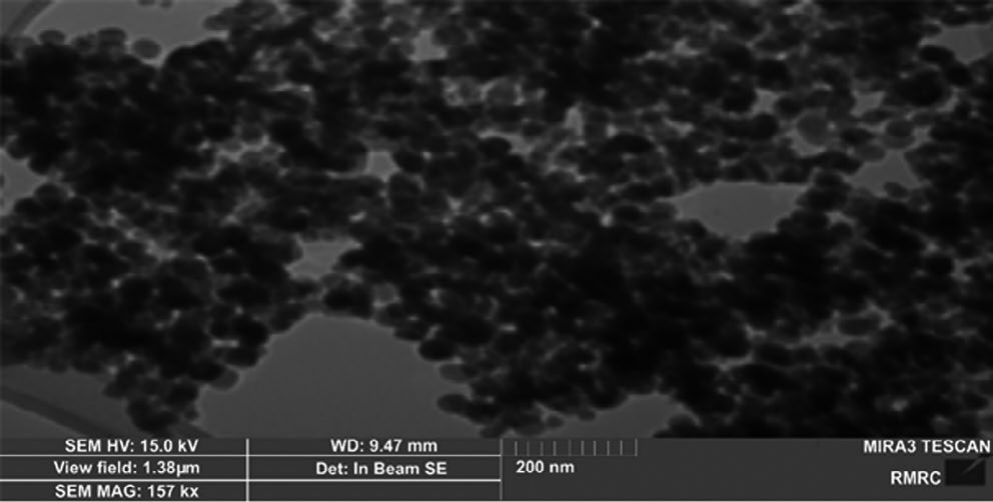
Scanning electron micrograph (SEM) image of TiO_2_‐NPs

**Figure 2:**
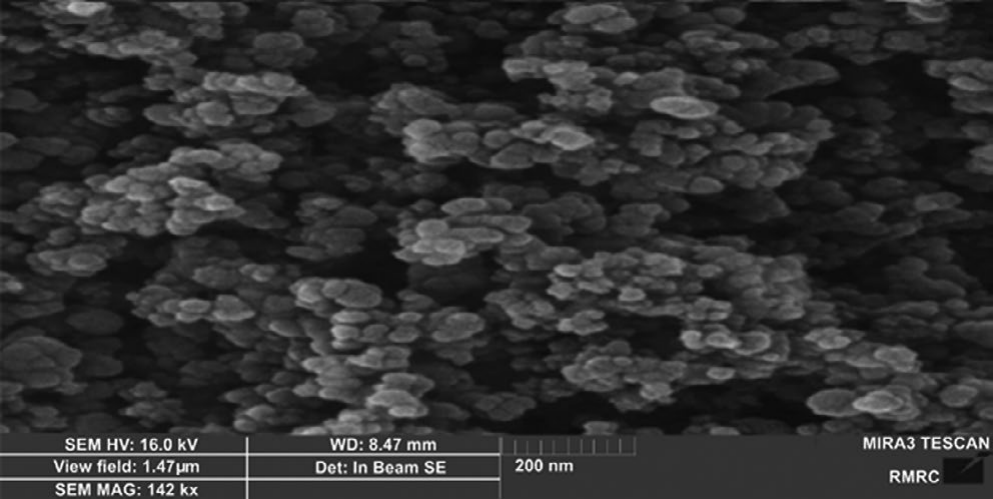
Scanning electron micrograph (SEM) of ZnO‐NPs

As shown in Figure 1, TiO2‐NPs have anatase form of 99% purity. The size of many nanoparticles is in the range of 10‐25 nm. The SEM image shows a uniform distribution of the nanoparticles. The TiO2‐NPs were spherical or rod‐shaped and composed of 80% anatase and 20% rutile (Figure 1). Figure 2 shows the ZnO‐NPs with a spherical structure of 99.8% purity. The size of most ZnO‐NPs is in the range of 10‐30 nm with an average of 20 nm. The SEM image shows a uniform distribution of the nanoparticles.

### 3.2 X‐ray diffraction patterns of TiO2‐NPs and ZnO‐NPs

**Figure 3:**
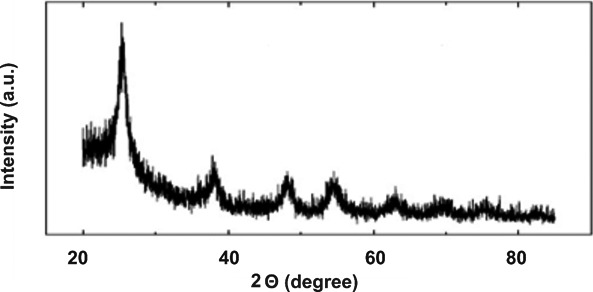
X‐ray diffraction pattern obtained from TiO_2_‐NPs

**Figure 4:**
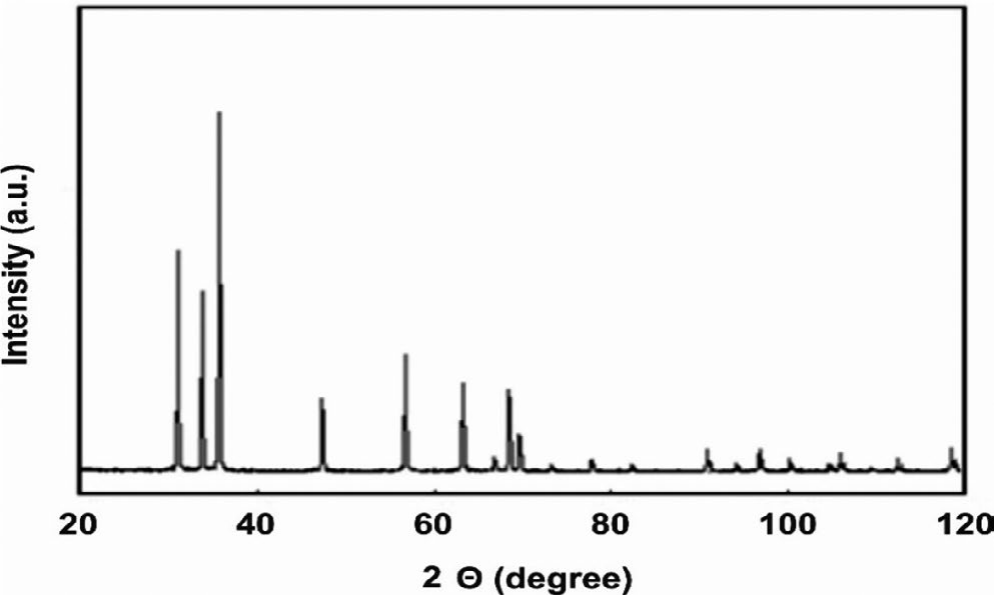
X‐ray diffraction pattern obtained from ZnO‐NPs

Figure 3 shows the X‐ray diffraction pattern of TiO2‐NPs in the anatase phase. Several sharp peaks are observed in the range 2Ɵ within 20‐80^°^. The identified peaks in the range 2Ɵ are 25.12^°^, 38.25^°^, 48.10^°^, 54^°^, 55.1^°^, 63^°^, 69.0^°^, 70.50^°^, and 75.10^°^. In the X‐ray diffraction pattern of ZnO‐NPs, several sharp peaks are observed in the range 2Ɵ in the range 20‐120^°^ (see Figure 4). These peaks are identified in the range 2Ɵ: 28.36^°^, 32.25^°^, 34.32^°^, 48.0^°^, 58.91^°^, 63.0^°^, 70.0^°^, and 71.20^°^.

### 3.3 Antifungal effects of TiO_2‐_NPs and ZnO‐NPs compared to AmB on different *Candida* species

**Figure 5:**
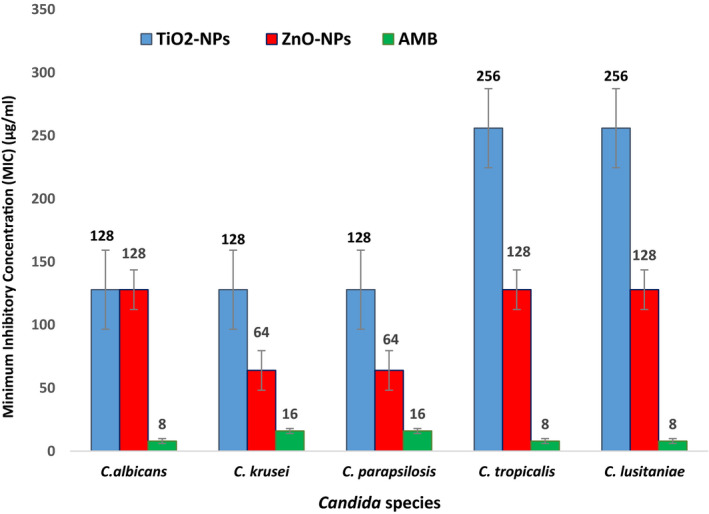
Minimum inhibitory concentration (MIC) values of TiO_2_‐NPs and ZnO‐NPs compared to amphotericin B against *Candida* spp (µg/mL)

**Figure 6:**
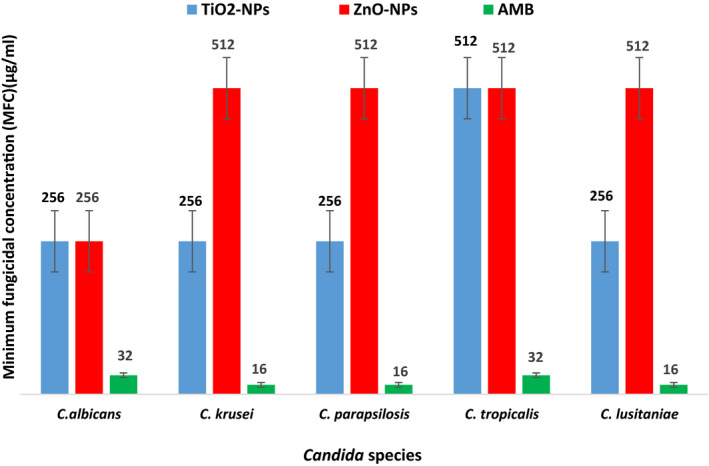
Minimum fungicidal concentration (MFC) values of TiO_2‐_NPs and ZnO‐NPs compared to amphotericin B against *Candida* spp (µg/mL)

Susceptibility of different *Candida* species to TiO_2_‐NPs and ZnO‐NPs compared to AmB is shown in Figures 5 and 6. As seen in Figures 5 and 6, MIC and MFC values of TiO_2_‐NPs against *Candida* spp. were 128‐256 µg/mL and 256‐512 µg/mL, respectively. The range of MIC and MFC of ZnO‐NPs was 64‐128 µg/mL and 256‐512 µg/mL, respectively. MIC and MFC values of AmB were 8‐16 µg/mL and 16‐32 µg/mL, respectively.

As shown in Figures 5 and 6, the lowest MIC of TiO2‐NPs in *C albicans*, *C krusei,* and *C parapsilosis* was 128 µg/mL and the highest MIC of TiO2‐NPs in *C lusitaniae* and *C tropicalis* was 256 µg/mL. Moreover, the results indicated that the highest MFC of TiO2‐NPs for *C tropicalis* was 512 µg/mL and the lowest MFC of TiO2‐NPs in *C parapsilosis*, *C lusitaniae*, *C krusei,* and *C albicans* was 256 µg/mL. The minimum amount of ZnO‐NPs required for the growth inhibition of *C parapsilosis* and *C krusei* was 64 µg/mL and for *C lusitaniae*, *C albicans,* and *C tropicalis* was 128 µg/mL. The lowest MIC of ZnO‐NPs was observed for *C parapsilosis* and *C krusei*, and the highest MIC values were obtained for *C lusitaniae, C albicans,* and *C tropicalis*.

The lowest MIC value of AmB in *C lusitaniae*, *C albicans,* and *C tropicalis* was 8 µg/mL, and the highest MIC value of AmB in *C parapsilosis,* and *C krusei* was 16 µg/mL. The results revealed that the highest MFC of AmB in *C tropicalis* and *C albicans* was 32 µg/mL and the lowest MFC of AmB in *C parapsilosis*, *C lusitaniae,* and *C krusei* was 16 µg/mL.

In the present study, using the agar well diffusion method, the diameters of zones of inhibition of five different *candida* spp in confronting various concentrations of TiO_2_‐NPs, ZnO‐NPs and AmB were measured. The highest zone of inhibition of the TiO_2_‐NPs at concentrations 8192 and 4096 μg/mL on all tested *Candida* spp was ≥60 mm. No growth inhibition in *Candida* species was observed in concentrations ˂512 μg/mL of TiO_2_‐NPs.

The largest inhibition zone of ZnO‐NPs at concentrations of 8192 μg/mL on *C parapsilosis* was obtained by the size equal to 20 mm. No growth inhibition was observed at concentrations 2048 μg/mL of ZnO‐NPs for *C parapsilosis, C tropicalis,* and *C krusei*, and at concentrations ˂2048 μg/mL of ZnO‐NPs for all tested *Candida* spp. The maximum of zones of inhibition on *C albicans*, followed by *C krusei* and *C parapsilosis* produced by concentrations of 128 μg/mL of AmB. Totally, it suggested that the antifungal activities of TiO_2_‐NPs, ZnO‐NPs, and AmB increased with increase in concentrations. Diameters of zones of inhibition of TiO2‐NPs, ZnO‐NPs, and AmB at various concentrations against different *Candida* species (mm) are shown in Tables 1, 2, 3, respectively.

**Table 1:** Zone of inhibition of TiO2‐NPs at various concentrations against different *Candida* species (mm)

Concentrations of TiO2‐NPs (μg/mL)							
*Candida* species	8192	4096	2048	1024	512	<512	Negative control
*C parapsilosis*	>60	>60	30	20	0	0	0
*C lusitaniae*	>60	>60	>60	20	7	0	0
*C krusei*	>60	>60	>60	23	10	0	0
*C tropicalis*	>60	>60	>60	20	7	0	0
*C albicans*	>60	>60	>60	15	7	0	0

**Table 2:** Zone of inhibition of ZnO‐NPs at various concentrations against different *Candida* species (mm)

Concentrations of ZnO‐NPs (μg/mL)							
*Candida* species	8192	4096	2048	1024	512	<512	Negative control
*C parapsilosis*	20	10	0	0	0	0	0
*C lusitaniae*	10	7	6	0	0	0	0
*C krusei*	10	7	0	0	0	0	0
*C tropicalis*	10	7	0	0	0	0	0
*C albicans*	10	9	7	0	0	0	0

**Table 3:** Zone of inhibition of amphotericin B at various concentrations against different *Candida* species (mm)

Concentrations of AmB (μg/mL)						
*Candida* species	128	64	32	16	8	Negative control
*C parapsilosis*	28	23	21	19	15	0
*C lusitaniae*	25	20	18	17	15	0
*C krusei*	29	23	20	19	17	0
*C tropicalis*	25	22	20	19	17	0
*C albicans*	30	25	20	19	17	0

### 3.4 MTT assay results

**Figure 7:**
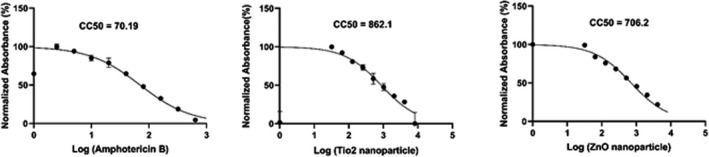
Survival rate of mouse macrophages J/774 exposed to TiO_2_‐NPs and ZnO‐NPs compared to amphotericin B

Survival rate of mouse macrophages J/774 exposed to TiO_2_‐NPs and ZnO‐NPs compared to AmB is shown in Figure 7. The MTT results showed that the CC50% were for ZnO‐NPs 706.2 μg/mL, for TiO2‐NPs 862.1 μg/mL, and for AmB 70.19 μg/mL, respectively. Our finding indicated that the rate of cytotoxicity increased with increasing concentration. The CC50% of AmB was significantly lower than ZnO‐NPs and TiO2‐NPs (P < .05). The cytotoxicity ratio ranking on mouse macrophages J/774 was TiO2‐NPs* > *ZnO‐NPs* > *AmB.

## 4 DISCUSSION

In the recent decades, the prevalence of opportunistic diseases such as fungal infections in immunocompromised patients has been increased. Candidiasis is the major common invasive fungal infection in human.^8^ Due to the side effects of antifungal drugs and the development of resistant fungal species and therapeutic failures, attention has been drawn to the use of novel antifungal compounds with fewer side effects.^18^ One of these novel compounds is nanoparticles. The nanoparticles are very small substances <100 nm in size that have various physical and chemical properties, act on living cells at the nanoscale and initiate different effects.^19^ Of all nano‐sized materials, metal oxide nanoparticles are the most attractive. Some of these metal nanoparticles are considered in medicine and pharmaceutical sciences due to their unique properties. ZnO‐NPs, magnesium oxide, and TiO2‐NPs are examples of these nanoparticles.^20^, ^21^

The primary aim of this study was to evaluate the antifungal effects of TiO2‐NPs and ZnO‐NPs compared to AmB on different *Candida* spp. Our findings showed that the TiO_2_‐NPs and ZnO‐NPs had antifungal potentials against pathogenic *Candida* spp. and could stop the growth of all tasted *Candida* spp at different concentrations, although their inhibitory effect is less than AmB. The results of Karimiyan et al study were consistent with those of the present study. They showed that ZnO‐NPs and copper oxide (CuO) nanoparticles exerted their inhibitory effect at a higher concentration compared to AmB; also, magnesium and silicon nanoparticles had no inhibitory effect on *C albicans*.^22^

Moreover, Memarian et al demonstrated that gold nanoparticles exerted their inhibitory effect on resistant strains of *C albicans* at a higher concentration as compared with FLC and conjugated FLC.^23^ Dananjaya et al concluded that significant changes occurred in the external morphology of *C albicans* after treatment with both types of ZnO‐NPs due to the damage to the fungal cell membrane. In addition, the cytotoxicity of both nanoparticles was compatible with the increase in the concentration of Hep2 cells and *C albicans*.^24^ In another study, chemically synthesized TiO2‐NPs exhibited higher anti‐candidal property compared with FLC.^25^

In the present study, the inhibitory effect of the ZnO‐NPs occurred at MIC = 128 μg/mL for *C albican*, while MIC of ZnO‐NPs and ZnO‐chitosan nanocomposites on *C albican* was 200 μg/mL and 75 μg/mL, respectively.^24^ In the study concluded by Grabchev et al, copper complex molecules and benzofuran‐cyclam had a greater antibacterial and antifungal effect than copper free ligand.^26^

The secondary aim of the present study was to compare the cytotoxic effect of TiO2‐NPs and ZnO‐NPs with AmB. Our findings indicated that the rate of cytotoxicity increased with increasing concentration. The CC50% of AmB was significantly lower than ZnO‐NPs and TiO2‐NPs.

Previous studies have shown the cytotoxic activity of different nanoparticles against various cell lines. Ghadimi et al reported that the toxicity of the nanoparticles was dose‐dependent at 24 hours and the highest cytotoxic effects were observed at concentrations of 50 and 100 μg/mL and IC50 equal to 7.14 μg/mL.^27^ In the current study, the CC50% of TiO2‐NPs was significantly higher on mouse macrophages J/774, than those reported in the study performed by Ankita Chatterjee et al,^28^ who reported that the TiO2‐NPs at a concentration of 200 µg/mL exhibited cytotoxic activity against the Mg 63 osteosarcoma cell lines. The cytotoxic effects of ZnO‐NPs on lung cancer cell line A549 occurred at 33‐37 μg/mL.^29^ Different nanoparticles have completely different antifungal and cytotoxic effects, which can be explained by the type, size, and synthesis mode of the nanoparticles as well as the studied *Candida* species.

## 5 CONCLUSION

Our finding showed that the TiO_2_‐NPs and ZnO‐NPs had antifungal potentials against pathogenic *Candida* spp. and could inhibit the growth of all tested *Candida* spp. However, its antifungal properties were significantly less than those of AmB. MTT assay results revealed that the rate of cytotoxicity increased with increasing concentrations. Finally, the CC50% of AmB was significantly lower than ZnO‐NPs and TiO2‐NPs.

## CONFLICT OF INTEREST

The authors declare no competing interests.

## AUTHORS CONTRIBUTIONS

SS and PGHA developed the study concept and design. SHA collected the data. SS analyzed and interpreted the data. SS wrote the article. PGHA and SS revised and edited the article. All authors read and approved the final article.

## ETHICAL APPROVAL

The study was evaluated and approved by the Ethics Committee of the Kerman Medical University and Kerman Research Council (IR.KMU.REC.1397.542).
